# Belladonna *vs.* Opium; Administration of Chloroform during Sleep; Unusual Number of Worms

**Published:** 1875-04-15

**Authors:** J. J. Schneck

**Affiliations:** Mt. Carmel, Ill


### MEDICAL EXAMINER.

A

*Semi-Monthly Journal of Medical Sciences.*

EDITED BY N. S. DAVIS, M.D., AND F. H. DAVIS, M.D.

No. VIII. CHICAGO, APRIL 15, 1875. VOL. XVI.

### Original Communications.

### BELLADONNA VERSUS OPIUM; ADMINISTRATION OF CHLOROFORM DURING SLEEP; UNUSUAL NUMBER OF WORMS.

NOTES FROM PRACTICE, BY J. SCHNECK, M.D., MT. CARMEL, ILL.

IN his valuable paper in the EXAMINER of February 15th, 1874, on "The Antagonistic Effects of Belladonna and Opium," Dr. Wardner, after having examined the literature on the subject, deprecates the fact that those cases in which the results have been unfavorable, have not been published. He has been able to find but one reported case (that of Dr. J. P. Chesney) of opium-poisoning, in which belladonna was given as an antidote, that has terminated unfavorably, the subject in this case being an infant.

However, in the same number of the EXAMINER, under the head of "Transactions of the Chicago Society of Physicians and Surgeons," are two other fatal cases of opium-poisoning reported in which belladonna was given, one by Dr. Etheridge, and another by Dr. Wilder. In both cases the antidote was given until the physiological effect (dilatation of the pupils) was produced.

The following case, it is thought, will be of interest in this connection, as adding a fourth fatal case:

BELLADONNA VERSUS OPIUM.

, Oct. 9, 1871, at 5 p. m., was called to see W. G——, physician, aged near forty years; of nervous temperament; had for years been an immoderate user of alcoholic stimulants. Upon my arrival at his office (less than a square's distance from my own), found him in profound coma, breathing sterorously, deeply suffused condition of the skin, pupils very small, sensibility almost entirely gone, consciousness rapidly failing, but, after severe shaking and loud talking, aroused him sufficiently to tell me indistinctly, "I have taken thirty grains of morphine; I did it on purpose; I knew what I was doing." A note was found on his table containing a similar statement, where were also found an empty morphine bottle and a goblet. Consciousness and sensibility now rapidly disappeared. From what I can learn from his brother, the poison had not been taken over thirty minutes when I first saw him. After all efforts to produce vomiting had failed, galvanism, artificial respiration, stimulants, rubefacients, etc., were diligently applied. In addition, hypodermic injections of atropiæ sulphas were given at intervals of thirty minutes, until full dilatation of the pupils was produced, which effect was produced by about 7 p. m., two hours after he was first seen, three injections having been given. This effect was kept up during the next seventeen hours by two subsequent injections, the total amount of atropia given being about one and one-half grains. During the time from the eighth to the tenth hours after the poison was taken, there appeared to be improvement, sensibility and consciousness partially returning, but not sufficiently for conversation. After this time he again grew worse, and at 12 m., Oct. 10th, he died, nineteen hours after the morphine was taken. Although the atropia in this case did not save life, yet I think the improvement during the time from the eighth to the tenth hours is plainly to be accredited to it.

### ADMINISTRATION OF CHLOROFORM DURING SLEEP.

April 28th, 1873, was called to see J. W——, a rugged-framed but anæmic boy of five years. Examination revealed a large polypus of the vesicular variety in the left naris. Upon making an attempt to extract it, he became so badly frightened that I had to desist. Called the next morning at six o'clock, and found him asleep. I rolled a handkerchief into the shape of a bird's nest, in which I poured about one drachm of chloroform; this was at first held about six inches from the mouth and gradually brought nearer until total anæsthesia was produced. I now extracted the polypus, and did not even appear to disturb him of his sleep.

### UNUSUAL NUMBER OF WORMS.

Was called Feb. 13th, 1875, to see R. W——, a healthy farmer boy fourteen years old, slender form. Was given the following history: He had been attending school during the past three months; when out of school, had been doing such work as is usually done on a farm during the winter season; that he had during this time felt well; had been working hard all this day until about 5 p. m., when he was attacked with pain in the bowels, nausea, and finally vomiting. I saw him at 8 p. m., and found him some easier, but still complaining of pain in the abdomen, which, on examination, I found to be very hard and uneven to the touch. Otherwise could detect nothing wrong. Left him seven powders, each consisting of two grains each of santonin and calomel; four to be given this evening, one every two hours; at 6 the next morning a full dose of castor oil. The remaining three powders to be given two days afterward in the same way.

Feb. 18th. Heard from my patient to-day, and he is reported by his father, a man of undoubted veracity, to have passed 188 round worms (ascaris lumbricordes), all of good size. The patient says: "I am feeling as well as usual, and feel as if I was much taller."

